# Pathology of Tinnitus and Hyperacusis-Clinical Implications

**DOI:** 10.1155/2015/608437

**Published:** 2015-10-26

**Authors:** Aage R. Moller, Richard Salvi, Dirk De Ridder, Tobias Kleinjung, Sven Vanneste

**Affiliations:** ^1^The University of Texas, Richardson, TX 75080, USA; ^2^University of Buffalo, Buffalo, NY 14214, USA; ^3^University of Otago, Dunedin 9016, New Zealand; ^4^University of Zürich, Zürich, CH 8091, Switzerland

Not long ago, tinnitus and hyperacusis were considered intractable symptoms and the lack of interest and shortage of research in diseases with these symptoms would have made publishing a special issue on tinnitus and hyperacusis nearly impossible. During the past two decades, there has been an explosion of research on tinnitus and incremental growth on hyperacusis, a condition associated with hearing loss, autism, migraine, closed head injuries, Williams syndrome, fibromyalgia, and other sensory hypersensitivity disorders. Prior to 1980, a search of PubMed turned up fewer than 25 publications with tinnitus in the title (Figure 1); the situation for hyperacusis was even more dismal with less than 5 publications in 1980 and only 19 in 2014.

This increase in publications reflects a large increase in research made possible by new hypotheses about the pathology of these diseases, advances in neuroscience in general, and new technological approaches. The increase in research funding by private philanthropic organizations such as the American Tinnitus Association, the Tinnitus Research Consortium, the Tinnitus Research Initiative, and Action on Hearing Loss has been essential for the progress in understanding of tinnitus and hyperacusis and the treatment of these disorders. Research grants from governmental agencies have also contributed to these advances in research regarding tinnitus and hyperacusis.

The incentive for this special issue was the tremendous personal, social, and financial costs associated with tinnitus and hyperacusis. For those suffering from severe or debilitating tinnitus or hyperacusis, the psychosocial and emotional costs can be enormous. While tinnitus and hyperacusis can affect anyone, young or old, those serving in the military are at a higher risk than nonmilitary people. Roughly 50% of combat personnel in the Gulf War developed tinnitus where exposure to intense noise and stress were likely the major contributing factors. Tinnitus ranks as the #1 service-connected disabilities in the Veterans Health Care System with compensation costs $1.2 billion for the year 2012, projected to reach $3 billion for the year 2017.

The completion of this special issue is a testament to the tremendous efforts by research groups around the world to develop a better understanding of the neural mechanisms underlying tinnitus and hyperacusis and to develop better and more effective therapies. This special issue combines association studies (tinnitus and sleep, tinnitus and headaches, tinnitus and interoceptive awareness, mastoid pneumatization, and pulsatile tinnitus), diagnostic studies (how to measure hyperacusis, the relevance of high-frequency hearing loss in tinnitus), and treatment studies (coordinated reset acoustic stimulation, repeated rTMS sessions).

A few highlights from the accepted papers in this special issue are discussed below.

- Hearing loss, which reduces the neural input to the central auditory system, is thought to be one of the major triggers for inducing tinnitus and aberrant neural activity within the brain; however, many people with tinnitus have normal hearing thresholds within the conventional audiometric range (0.25–8 kHz). The work of V. Vielsmeier et al. shows that many people with tinnitus who have what is regarded to be normal hearing have elevated hearing thresholds above 8 kHz, which are strongly correlated with the laterality of the tinnitus. The take home message is that high-frequency audiometry should be an integral part of a comprehensive tinnitus assessment.
- Some evidence suggests that the air spaces within the temporal bone (pneumatization) may contribute to the severity of pulsatile tinnitus. Using imaging techniques to quantify pneumatization, W. Liu et al., however, found little correlation between the severity of tinnitus and the degree of pneumatization.
- While many people with tinnitus have hearing loss, not everybody who has hearing loss has tinnitus, a result that supports other findings that show that tinnitus is a multifactorial disease. The article by B. Langguth et al. present evidence that tinnitus and headache may be pathologically linked, consistent with earlier research linking tinnitus and hyperacusis to migraine.
- Sleep disturbances are common in people with tinnitus but the relationship between sleep disturbance and the severity of a person's tinnitus has been unclear. M. Schecklmann et al. report that tinnitus distress is highly correlated with sleep disturbances.
- Over the past decade, many new and promising therapeutic approaches for treating tinnitus have emerged. Many different sound therapies designed to modify neural activity in the brain have been developed and remain to be validated. The exciting paper by C. Hauptmann et al. suggests that acoustic coordinated reset neuromodulation could become a therapeutic strategy for treating patients with chronic tonal tinnitus. Even though the lack of a control group does not permit showing real efficacy, the promising results of this open label study demonstrate that further controlled studies are warranted.
- Another approach to treating people with tinnitus is repetitive transcranial magnetic stimulation (rTMS). One of the main problems with published studies of the use of rTMS to treat people with tinnitus is the small effect size and the fact that the effect of rTMS in tinnitus is limited in time. In a paper in this issue A. Lehner et al. demonstrate that repeating the rTMS sessions seems to be beneficial when the tinnitus distress worsens after waning of the rTMS effect.
- The Hyperacusis Questionnaire is a tool used by clinicians to evaluate hyperacusis symptoms in tinnitus patients. Factor analysis of data obtained by K. Fackrell et al. suggests that only 10 items and two factors (attentional and social) in the Hyperacusis Questionnaire may be a more appropriate approach for assessing hyperacusis instead of the current 12 items and 3 factors (emotional, attentional, and social).
- Furthermore, it was shown by M. Schecklmann et al. that using only 2 questions can give a good hint at whether hyperacusis is present: (1) Do you have a problem tolerating sounds because they often seem much too loud? (2) Do sounds cause you pain or physical discomfort?
- P. Lau et al. demonstrate that tinnitus is unrelated to interoceptive awareness but that people with tinnitus tend to overestimate physical changes in comparison to people who do not have tinnitus.

In summary, special issues like this, covering clinical, diagnostic, and treatment aspects of tinnitus and hyperacusis, remain highly needed to continue the quest for finding better and more effective ways to treat these elusive symptoms. Only a better understanding of the causes of both tinnitus and hyperacusis and their pathology can pave the way to reaching this goal.

*Aage R. Moller* *Richard Salvi* *Dirk De Ridder* *Tobias Kleinjung* *Sven Vanneste*

## Figures and Tables

**Figure 1:**
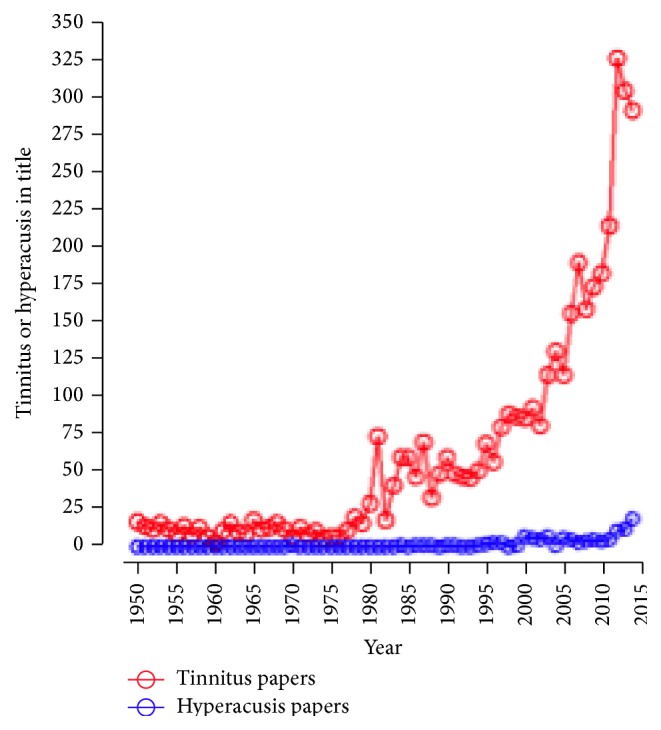
A search of PubMed shows an exponential increase of publications related to tinnitus over the last 20 years, while research related to hyperacusis has been mainly overlooked.

